# Establishment of an Artificial Neural Network Model Using Immune-Infiltration Related Factors for Endometrial Receptivity Assessment

**DOI:** 10.3390/vaccines10020139

**Published:** 2022-01-18

**Authors:** Bohan Li, Hua Duan, Sha Wang, Jiajing Wu, Yazhu Li

**Affiliations:** 1 Department of Minimally Invasive Gynecologic Center, Beijing Obstetrics and Gynecology Hospital, Capital Medical University, Beijing Maternal and Child Health Care Hospital, Beijing 100006, China; 112020010200@ccmu.edu.cn (B.L.); wangsha1020@ccmu.edu.cn (S.W.); 122020010416@mail.ccmu.edu.cn (Y.L.); 2 Department of Biochemistry and Molecular Biology, School of Basic Medical Sciences, Capital Medical University, Beijing 100069, China; jingjing@ccmu.edu.cn

**Keywords:** immune infiltration, macrophage polarization, weighted gene co-expression network, endometrial receptivity assessment, artificial intelligence

## Abstract

Background: A comprehensive clinical strategy for infertility involves treatment and, more importantly, post-treatment evaluation. As a component of assessment, endometrial receptivity does not have a validated tool. This study was anchored on immune factors, which are critical factors affecting embryonic implantation. We aimed at establishing novel approaches for assessing endometrial receptivity to guide clinical practice. Methods: Immune-infiltration levels in the GSE58144 dataset (*n* = 115) from GEO were analysed by digital deconvolution and validated by immunofluorescence (*n* = 23). Then, modules that were most associated with M1/M2 macrophages and their hub genes were selected by weighted gene co-expression network as well as univariate analyses and validated using the GSE5099 macrophage dataset and qPCR analysis (*n* = 19). Finally, the artificial neural network model was established from hub genes and its predictive efficacy validated using the GSE165004 dataset (*n* = 72). Results: Dysregulation of M1 to M2 macrophage ratio is an important factor contributing to defective endometrial receptivity. M1/M2 related gene modules were enriched in three biological processes in macrophages: antigen presentation, interleukin-1-mediated signalling pathway, and phagosome acidification. Their hub genes were significantly altered in patients and associated with ribosomal, lysosomal, and proteasomal pathways. The established model exhibited an excellent predictive value in both datasets, with an accuracy of 98.3% and an AUC of 0.975 (95% CI 0.945–1). Conclusions: M1/M2 polarization influences endometrial receptivity by regulating three gene modules, while the established ANN model can be used to effectively assess endometrial receptivity to inform pregnancy and individualized clinical management strategies.

## 1. Introduction

Endometrial receptivity is a complex process that enables embryonic attachment, invasion, and development. For healthy females, during the secretory phase, the window of implantation (WOI) lasts from 3 to 6 days. In certain inflammatory or anatomical cases, this window can be narrowed or displaced to inhibit normal implantation, resulting in infertility or loss of pregnancy [1]. Therefore, a receptive endometrium is a prerequisite for successful embryonic implantation. Defective endometrial receptivity is often associated with repeated implantation failure (RIF), embryonic loss and unexplained infertility. However, a lack of understanding of defective endometrial receptivity has led to poor diagnosis and treatment. The current diagnostic tests for endometrial receptivity disorders include sonography, histopathology, and electron microscopy of endometrial cell morphologies. However, these methods have a limited clinical guidance [2].

Immune status of the endometrium is closely associated with normal reproductive functions [3]. Trophoblast cells of implanted blastocyst invade the endometrium of the maternal uterus by forming the placenta. Then, endometrial mesenchymal cells undergo a decidual response (called decidualization) to establish an environment that is conducive for trophoblast invasion. Trophoblast implantation and formation of the placenta require appropriate maternal immune tolerance to the hemi allogeneic foetus. Embryonic implantation and decidual development are associated with significant changes in the number of leukocytes in the uterus [4]. During the early gestation period, natural killer (NK) cells and macrophages predominate. Macrophages, which account for approximately 20–30% of total infiltrating leukocytes, play an essential role in foetal tolerance, trophoblast invasion, and tissue as well as vascular remodelling [5]. Due to hormonal regulation and stress responses to clinical operation and dysbacteriosis, the immune environment of the endometrium is always in a dynamic balance. Therefore, endometrial receptivity assessment based on immune-related modules can predict implantation outcomes and predict pregnancy timing after a uterine cavity procedure (induced abortion, adhesiolysis, and polypectomy) or other therapies (antibiotics, hormones etc.). However, its value in assessing endometrial receptivity has not been conclusively determined. Decidual macrophages are characterized by an M1 (inflammatory) phenotype in the peri-implantation stage [6]. However, there exists a composite of M1 (inflammatory) and M2 (anti-inflammatory) decidual macrophages in the placental stage, shifting to a predominance towards M2 phenotype post-placentation, which is attributed to a potential stimulation by secretory factors [3]. Moreover, subgroups beyond the scope of conventional phenotyping are present. While decidual macrophages inhibit the occurrence of uterine infections in gestating women, the endometrium-macrophage crosstalk is involved in supporting normal placentation, given its contribution to implantation, placental development, immunoregulation, vascular remodelling, and tissue homeostasis [7]. The balance between M1 and M2 macrophages is important for normal pregnancy. However, the orientation of macrophage polarization and mechanisms affecting embryonic implantation have not been fully established [3,8]. Additionally, endometrial macrophage polarization during WOI in humans has not been well determined. Animal model-based studies and small sample sizes are some of the limitations for various studies. Furthermore, the application of macrophage polarization in clinical diagnosis or treatment of infertility is not well informed.

We found that a moderate increase in M1/M2 in WOI is beneficial for embryonic implantation. Further, weighted gene co-expression network analysis (WGCNA) was performed to establish gene modules, which maintains the integration of gene expression and biological interactions [9], and evaluated their associations with macrophage polarization [10]. The predictors selected in this approach have representative biological structures and functions. Previous evaluation models were established by linear equations. However, measurement errors across platforms and patients’ individualization often make it difficult to guarantee the consistency of results, which in turn affects repeatability of the method. By training the programs with specific patterns, the artificial intelligence approach can be used to guarantee the consistency of results across platforms and populations. The artificial neural network (ANN) is an artificial intelligence (machine learning) method that works similarly to the human brain [11]. Its incorporated feature variables, also known as predictors, input variables and covariates, are the input signals that inform pattern recognition. Each characteristic variable is weighted according to its clinical significance. The task is accomplished by dendrites in the biological nervous system. An activation function sums the weighted signals [12]. We performed WGCNA to identify immunological factors that were most associated with defective endometrial receptivity and used them to develop a fertility prognostic model through ANN. The results were validated via different platforms and populations to establish a more practical and reliable approach for endometrial receptivity assessment.

## 2. Materials and Methods

### 2.1. Datasets and Patient Selection

All datasets were selected from chip microarrays. The GSE58144 dataset, containing 43 defective endometrial receptivity (DER) patients (RIF) and 72 controls, was used for immune infiltration analyses, hub gene selection, and machine deep learning prognostic model establishment. The GSE5099 dataset, a three replicate measure dataset of gene expression matrices of M1 and M2 macrophages, was used to validate the association between hub genes and macrophage polarization. The GSE165004 dataset, which includes 48 DER patients (embryonic loss and unexplained infertility) and 24 controls, was used to validate hub gene expression differences and the machine deep learning prognostic model. Details of the microarrays are available in Supplementary Table S1.

**Figure 1:**
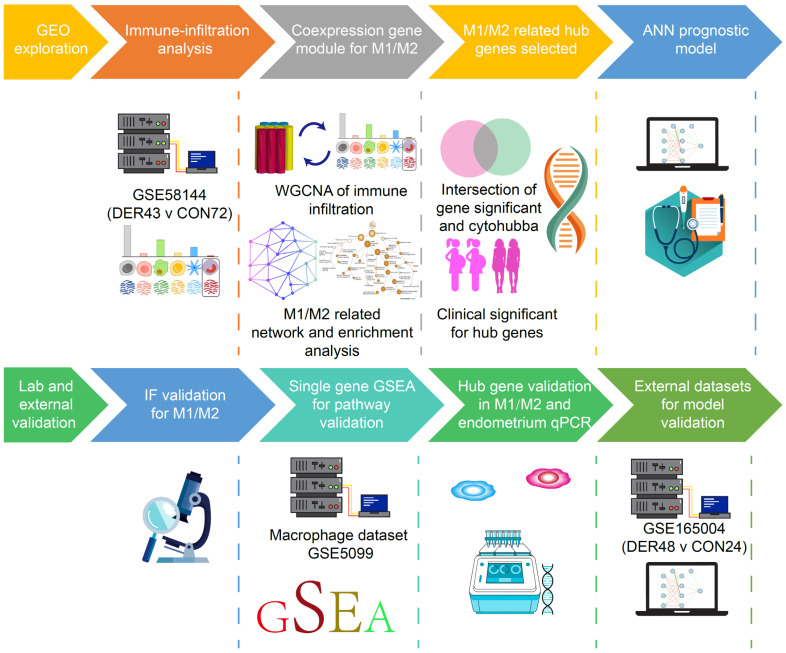
Schematic presentation of the study design. DER = defective endometrial receptivity, IF = Immunofluorescence, ANN = artificial neural network and GSEA = Gene-set enrichment analysis.

Validation of M1 and M2 macrophage ratios was performed using clinical samples. This study was ethically approved by the Research Ethics Committee of the Beijing Obstetrics and Gynecology Hospital. Experiments were performed (under protocol number 2017-KY-082-02) in accordance with the Helsinki Declaration of 1975 (revised in 2013). Patients that were eligible for hysteroscopy were required to sign an informed consent before surgery and follow up for 1 year. Samples from endometrial biopsies were acquired from the Beijing Obstetrics and Gynecology Hospital. The inclusion criteria for patients were (i.) those aged less than 40 years and desired a recent pregnancy; (ii.) those whose sex hormone levels, including follicle-stimulating hormone, luteinizing hormone, testosterone, estradiol and prolactin, were within normal ranges and (iii.) those without endometriosis, submucous myomas, active or a history of pelvic inflammatory disease or other medical comorbidities (hyperprolactinemia, thyroid disease etc) after procedures. Study participants in the control group (*n* = 10) had successful clinical pregnancies, while those in the DER group (*n* = 13) failed at having successful pregnancies during follow-up after procedures. Basic demographic characteristics for each group are presented in Supplementary Table S2. The flowchart of the study design is shown in Figure 1.

### 2.2. Processing of Primary Datasets

Pre-processing and normalization of microarray datasets based on raw data of the Affymetrix platform (GSE5099) were performed using the affy package in R version 3.6.2 [13], with the following methods: (i.) Robust multi-array average (RMA, for background correction) [14]; (ii.) Quantile (for normalization) [15]; (iii.) pmonly (perfect match correction) [16] and iv. Median polish (as a summary method) [17]. For the microarray datasets that were based on the Agilent platform (GSE58144 and GSE165004), Biobase and limma packages in R version 3.6.2 were used for pre-processing and normalization after the data had been converted to log (base 2). The RMA and normalizeBetweenArrays methods were respectively used for background correction and normalization [18]. Annotation files for different microarray platforms were downloaded from the NCBI GEO database [19].

### 2.3. Digital Deconvolution of Bulk Tissues

Cell-type deconvolution was performed using CIBERSORTx [20], an analytical tool developed by Newman et al. [21] to impute gene expression profiles and provide estimations of abundances of immune cell infiltration levels in mixed cell populations, using gene expression data. The LM22 gene signature matrix was used for assessing the infiltrations of 22 immune cell types. CIBERSORTx was run with batch correction and 100 permutations. Barplot and vioplot were established using the plot function in R version 3.6.2.

### 2.4. Immunofluorescence Assay

From each sample, 5 μm thick sections were prepared, dewaxed in xylene, dehydrated using graded alcohol and rinsed in distilled water. For antigen retrieval, sections were boiled in citric saline (10 mmol/L, pH 6.0) for half an hour. Then, samples were treated with 3% hydrogen peroxide solution for 25 min to block the activity of endogenous peroxidase, blocked using 3% bovine serum albumin (BSA, Servicebio, Wuhan, China) for 30 min at room temperature, after which they were incubated at 37 °C for 1 h with primary antibodies, including mouse anti-CD68 (ab201973, Abcam, dilution 1:200) and rabbit anti-CD86 (13395-1-AP, Proteintech, dilution 1:200) for M1 macrophages as well as mouse anti-CD68 and rabbit anti-CD86 (13395-1-AP, Proteintech, dilution 1:200) for M2 macrophages. Sections were rinsed thrice using phosphate-buffered saline (PBS) and stained using anti-rabbit-Alexa Fluor^®^ 488 (ab150073) and anti-mouse-Alexa Fluor^®^ 594 (ab150064) (both Abcam, Cambridge, UK) for 1 h at room temperature. Slides were mounted in the SlowfadeGold reagent containing DAPI (Thermofisher, Landsmeer, The Netherlands) and microscopically (Nikon, Nikon Corporation, Tokyo, Japan) evaluated. The percentages of M1 or M2 macrophages were respectively obtained by counting fluorescence positive cells and dividing them by DAPI signal points using Image J (Version 1.50b). For each specimen, counting was done in triplicate and the average calculated.

### 2.5. Establishment of the Weighted Gene Co-Expression Network

The network module was established via the WGCNA package in the R environment, version 3.6.2 using the GSE58144 dataset [22]. To minimize noise in the gene expression dataset, data was filtered as follows. First, Pearson correlation analysis was used to rank all genes according to their association with M1/M2. Correlations between genes and M1/M2 were established at a cut-off of *p* ≤ 0.05. To reduce the computational burden and enhance signals in our data, we used 2185 of the 5531 genes with the greatest variability ranked by the variance in our initial network construction with a cut-off value of var >0.05 [22]. By definition, module genes are highly connected (i.e., module genes tend to have a relatively high connectivity). Therefore, for module detection, restricting analysis to the most connected genes should not lead to a major loss of information. Then, we performed cluster analysis of 2185 genes in the 115 patients. The theory of the network construction algorithm has been previously described [23]. Briefly, for co-expression module identification, Pearson correlation matrices were first generated (average linkage method) for all pairwise genes. Then, an adjacency matrix was constructed using a “soft” power adjacency function, $a_{ij}={\left|cor\left(x_{i}\;y_{j}\right)\right|}^{\beta }$. Based on the scale-free topology criteria (R^2^ = 0.85), a β of 10 (10th power of the correlation) was selected. In WGCNA, a soft threshold parameter, beta, of the power function was used to ensure that the co-expression network (adjacency matrix) best approximates scale-free topology. This adjacency matrix was transformed into a topological overlap matrix to measure relative gene interconnectedness and proximity. Finally, gene co-expression modules corresponded to branches of the resulting hierarchical clustering tree (dendrogram). To ensure that genes in the analysed network exhibited sufficient correlations, we set the weight threshold of the co-expression network to 0.03. The visual network diagram was constructed using Cytoscape (version 3.4.0).

### 2.6. Enrichment Analysis of Functional Categories

The STRING v11.5 online tool (https://string-db.org/; accessed on 29 July 2021) was used for functional enrichment analyses of the gene module that was most associated with endometriosis, identified by WGCNA analysis. In the enrichment analysis, Gene Ontology (GO) terms (including Biological Process (BP), Cellular Component (CC), and Molecular Function (MF)) as well as the Kyoto Encyclopedia of Genes and Genome (KEGG) were used to evaluate functional categories and pathways for genes in the module. Correlations between genes and M1/M2 macrophage polarization were calculated using the cor function in R environment, version 3.6.2. Then, the GOplot package in the R environment, version 3.6.2, was used to visualize GO enrichment analysis results and their regulatory conditions. The formula for calculating the Z-score was: $z-score=\frac{\left(up-down\right)}{\sqrt{count}}$, which is a value that indicates whether GO terms are more likely to be decreased (negative value) or increased (positive value). Gene-set enrichment analysis (GSEA) was performed on the GSE5099 dataset using the “ClusterProfiler” (24) package in R. The Broad Molecular Signature Database (MSigDB v7.0) dataset in the Kyoto Encyclopedia of Genes and Genomes (KEGG) (c2.cp.kegg.v7.0.symbols) (both http://software.broadinstitute.org/gsea/msigdb; accessed on 21 May 2021) was used for pathway enrichment analyses. This database summarizes and presents specifically well-defined biological states and pathway processes. For statistical significance estimation, the GSEA program was run with 1000 permutations, and correlations between selected genes and other genes used to rank all genes.

### 2.7. Quantitative Real-Time PCR Analysis (qRT-PCR)

Total RNA was extracted from each sample using RNAiso Plus (Takara Bio Inc., Shiga, Japan) and quantified using a NanoDrop™ One Spectrophotometer (Thermo Fisher Scientific Inc. MA, Waltham, USA). The First-Strand cDNA Synthesis SuperMix Kit (AT301-3, EasyScript, Transgen, Beijing, China) was used for cDNA synthesis from 1 μg of total RNA per sample. The primers (Supplementary Table S3) used in this study were designed by Sangon Biotech Co., Ltd. Shanghai, China. PCR was performed using a LightCycler 480 PCR System (Roche, Germany) with the protocol for the SYBR Premix Ex TaqTM II (RR820A, TaKaRa, Shiga, Japan). Reaction conditions were: 95 °C for 30 s for initial denaturation, followed by 35 cycles of 5 s at 95 °C and 34 s at 60 °C [24]. Measurements were repeated thrice, and relative quantifications performed by the comparative CT (2^−ΔΔCT^) method.

### 2.8. Artificial Intelligence and Prognostic Evaluation

The ANN prognostic model was developed using the nnet package in R environment, version 3.6.2 [12]. To determine the number of units in the hidden layer, the GSE58144 dataset was used: $Average\;Accuracy=\overset{l}{\underset{i=1}{\sum }}\frac{TP_{i}+TN_{i}}{TP_{i}+FN_{i}+FP_{i}+TN_{i}}/l$ (TP = true positive, TN = true negative, FP = false positive, FN = false negative). To ensure maximum optimization of the prognostic model, NNET was run with 100 permutations in the GSE165004 dataset for verification [11].

To assess the efficacy of the prognostic model, Z tests were used to determine significant differences between the area under the receiver operating characteristic (ROCs) curves (AUCs) using the pROC package in R, version 3.6.2. Sensitivity, specificity, Youden index (YI), positive predictive value (PPV), and negative predictive value (NPV) were used to assess prognostic values of hub genes and the machine learning model. The PPV and NPV strongly depend on the prevalence. As previously reported, only about one-third of infertility patients eventually achieve clinical pregnancy through systemic therapy [25].

### 2.9. Routine Statistics

Comparisons of cell proportions and single gene expression levels between the two groups were performed by the Wilcoxon test. The results are expressed as median (range). Normally distributed continuous variables were analysed by the Student’s *t*-test, while the paired *t*-test was used for paired analysis, which described as mean (sd). All statistical analyses were performed using R, version 3.6.2. When the proportion of missing data is less than 10%, missing values were imputed using multiple imputation (mice package in R, version 3.6.2).

## 3. Results

### 3.1. Immune Infiltration Levels on Endometrial Receptivity

**Figure 2:**
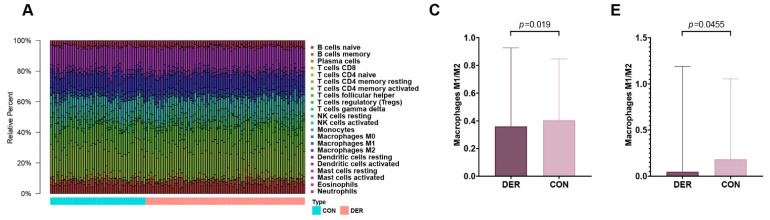

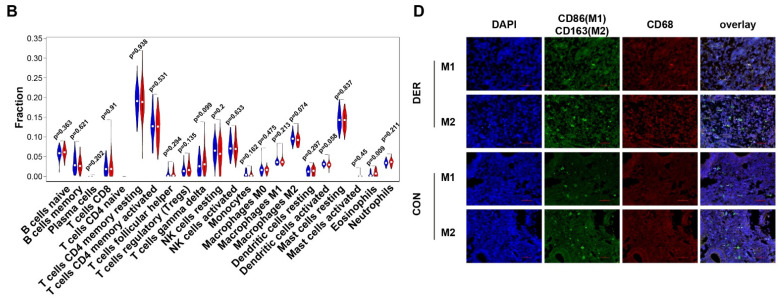
Immune infiltration analysis and M1 to M2 macrophage ratio. (**A**) Bar plots for 22 immune cells in endometrial mixed tissue samples. (**B**) Violin plots for immune cells in DER and control groups. Blue colour represents the DER group while the red colour represents the control group. (**C**) Comparisons of M1/M2 between the DER and control groups from the GSE58144 dataset. Values are presented as medians (ranges). (**D**) Immunofluorescence detection of M1 and M2 macrophages. CD86 (green) and CD68 (red) positivity represent M1. CD163 (green) and CD68 (red) positivity represent M2. White arrows in the overlay represent positive cells. (**E**) Immunofluorescence validation of M1/M2 comparisons in the DER (*n* = 13) and control (*n* = 10) groups. Values are presented as medians (ranges). DER = Defective Endometrial Receptivity.

Immune infiltration levels of 22 immune cells in the endometrial mixed tissue samples from the GSE58144 dataset were determined using the CIBERSORTx platform (Figure 2A). In different groups, differences in the percentage of immune cells were not significant, apart from eosinophils, which were present in deficient levels in the tissues (Figure 2B). Macrophage polarization is an important factor in endometrial receptivity. Therefore, we determined the ratio between M1 and M2 macrophages. It was established that differences in M1/M2 between DER and normal groups were significant (0.359 (0.251–0.927) vs. 0.404 (0.240–0.837), *p* = 0.019) (Figure 2C). Then, we obtained endometrial tissues from 30 patients within the mid-secretion phase for immunofluorescence detection. It was found that compared to the controls, the balance between M1 and M2 macrophages in the DER group was significantly altered (0.048 (0.012–1.190) vs. 0.183 (0.038–1.054), *p* = 0.0455) (Figure 2D,E).

### 3.2. Macrophage Polarization-Related Gene Module Functions

**Figure 3:**
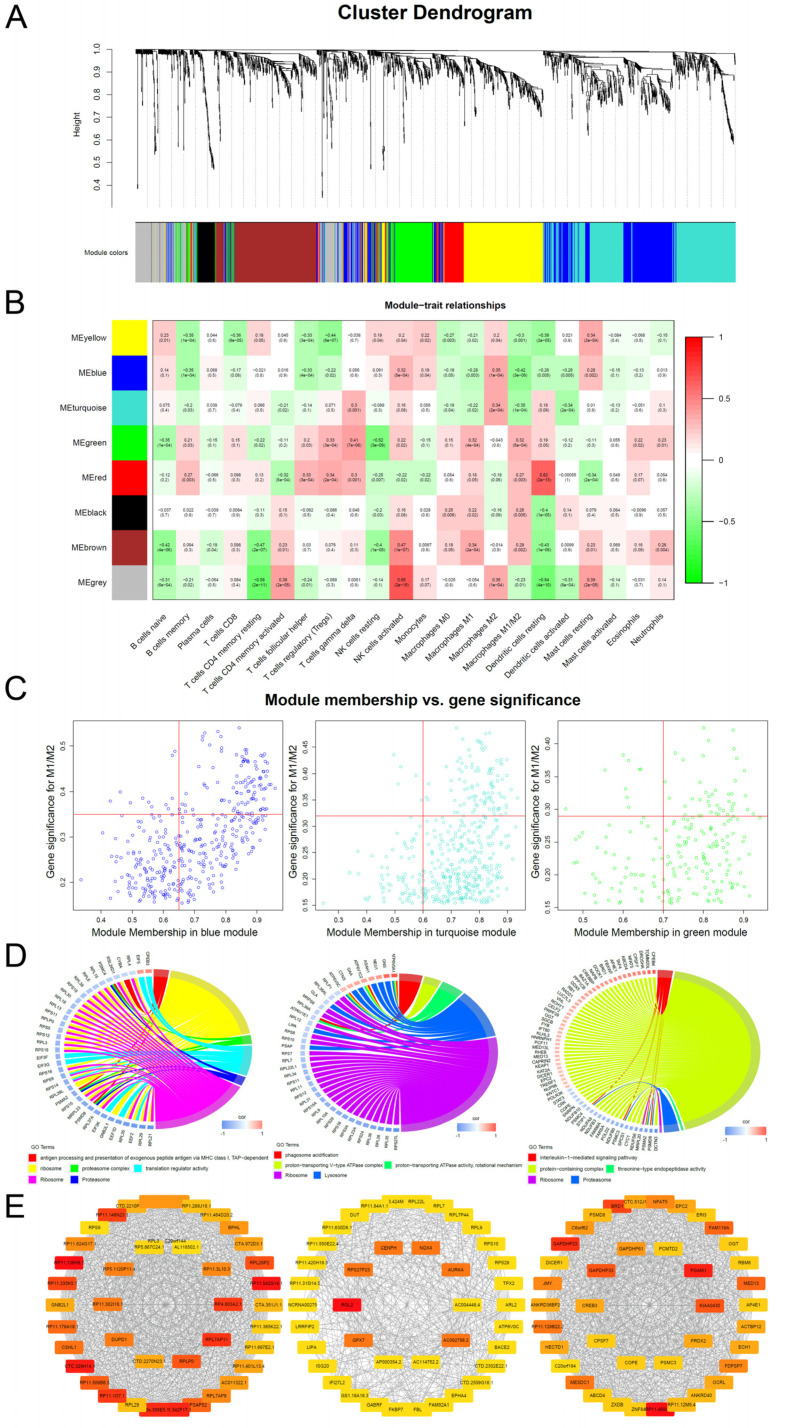
M1/M2 related WGCNA and module enrichment analyses. (**A**) Visual representations of the gene co-expression network. Hierarchical clustering of 2185 genes and visualization of gene module partitioning. Coloured bars (below) directly correspond to module (colour) designation for gene clusters. One can visualize where in the clustering dendrogram the gene modules are defined. (**B**) Heatmap showing the average genetic significance of each particular module across immune infiltration levels. (**C**) The correlation between module membership and gene significance in the three modules. (**D**) Enrichment analysis. The left side is the gene (the shade of the colour represents the gene’s fold change), while the right side are the different GO terms. Connected bands indicate that a gene is in its corresponding GO terms. (**E**) Co-expression network diagram. Top 40 hub genes were selected and visualized. Colour shades represent the number of connections.

As shown in Figure 3A, genes were clustered into different groups, referred to as modules. The GSE58144 gene set had 7 different gene modules with a high topological overlap. To distinguish among the modules, we allocated a colour to each module (including brown, black, Green, turquoise, red, blue, and yellow).

Then, we evaluated the pathological correlation for each module by assessing the overall correlation of module genes with clinical traits of immune infiltrations. The measure of gene significance was defined by the absolute value of the correlation between clinical factors and gene expression levels. The average genetic significance of a particular module is considered module significance (MS). As shown in Figure 3B, three modules (blue, turquoise, and green) exhibited significant correlations with M1/M2 (*p* = 3 × 10^−6^, 10^−4^, and 5 × 10^−4^), respectively, with moderate effect sizes (Pearson R = −0.42, −0.35, and 0.32, respectively). There were significant correlations between module membership of the genes within the module and gene significance between these genes and M1/M2 (*p*_blue_ = 1.6 × 10^−39^, *p*_turquoise_ =6.6 × 10^−15^, and *p*_green_ = 0.0093; Figure 3C). Then, we performed enrichment analysis of the three modules. The main biological processes of the three modules were the three aspects of macrophage functions (antigen processing and presentation of exogenous peptide antigens via MHC class I, TAP−dependent, FDR = 4.5 × 10^−10^; phagosome acidification, FDR = 6.2 × 10^−4^; interleukin-1-mediated signalling pathway, FDR = 1.13 × 10^−13^), while the corresponding GO-CC and GO-MF terms of the three were also different. KEGG enrichment analysis revealed that the three modules were respectively enriched in ribosomal, lysosomal, and proteasomal pathways (Figure 3D).

### 3.3. Selection and Verification of Hub Genes Associated with M1/M2

**Figure 4:**
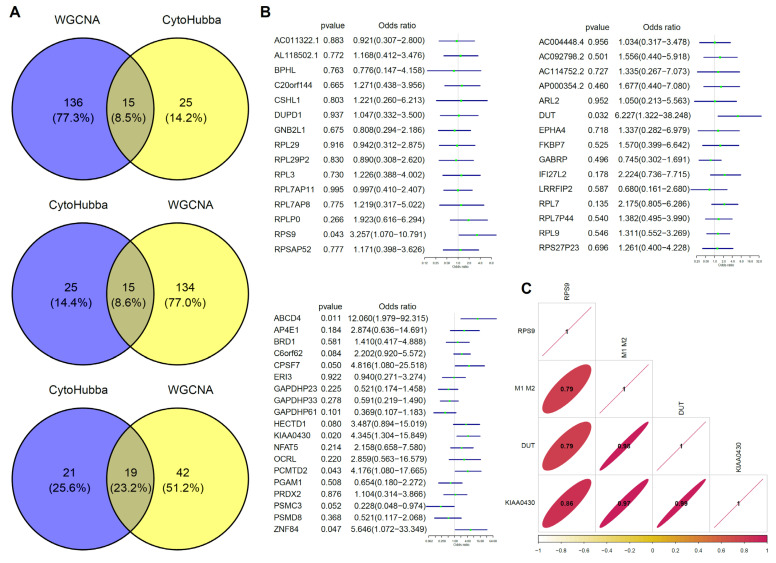

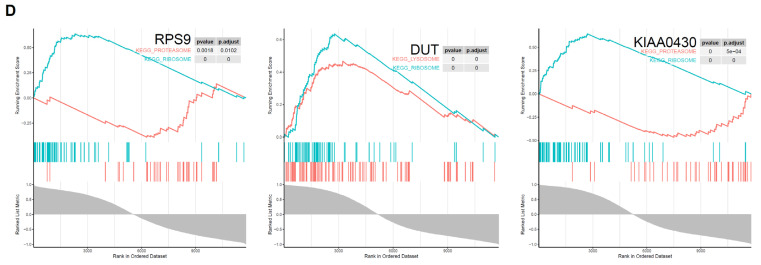
Hub gene selection and verification. (**A**) Venn plot for gene screening. Intersections between modules and hub genes calculated by CytoHubba were selected. (**B**) Univariate analysis of screened genes. RPS9 (OR (Odd rated) = 3.26 (95% CI 1.07–10.97)), DUT (OR = 6.23 (95% CI 1.32–38.25)), and KIAA0430 (OR = 4.35 (95% CI 1.30–15.85)) genes showed significant benefits for fertility. (**C**) Verification of correlation among hub genes and M1 and M2 macrophages. (**D**) Pathway validation of hub genes in macrophage polarization. RPS9 related genes shows an enrichment in ribosomal and proteasomal pathway (*p*_adjust_ = 0 and 0.0102), DUT in ribosomal and lysosome pathway (*p*_adjust_ = 0 and 0), and ribosomal and proteasomal (*p*_adjust_ = 0 and 5 × 10^−4^).

Due to differences in biological roles of the three gene modules and high levels of consistency in expressions among genes within the modules, we selected three hub genes in each of the modules. First, we selected genes that were most associated with modules and M1/M2 based on the median of the membership in the module and gene significance for M1/M2 (Figure 3C). Then, we screened the hub genes in the network based on the number of connections between nodes. CytoHubba was used to identify the top hub genes (Figure 3E). Intersections between the two sets of hub genes were established. Intersections for the genes in blue, turquoise, and green modules were 15, 15, and 19, respectively (Figure 4A). Finally, univariate analysis revealed that RPS9, DUT, and KIAA0430 genes were significantly associated with fertility in the blue, turquoise, and green modules (Figure 4B).

Then, we selected M1 and M2 macrophage datasets in GSE5099 to verify the associations between the above screened genes and macrophage polarization. As shown in Figure 4C, RPS9, DUT, and KIAA0430 genes exhibited high associations with M1 and M2 macrophages (0.79, 0.98, 0.97). Subsequently, GSEA results for the three genes were consistent with our previous findings, that is ribosomal (RPS9, DUT and KIAA0430), lysosomal (DUT), and proteasomal (RPS9 and KIAA0430), respectively (Figure 4D).

### 3.4. The Artificial Neural Network Prognostic Model

**Figure 5:**
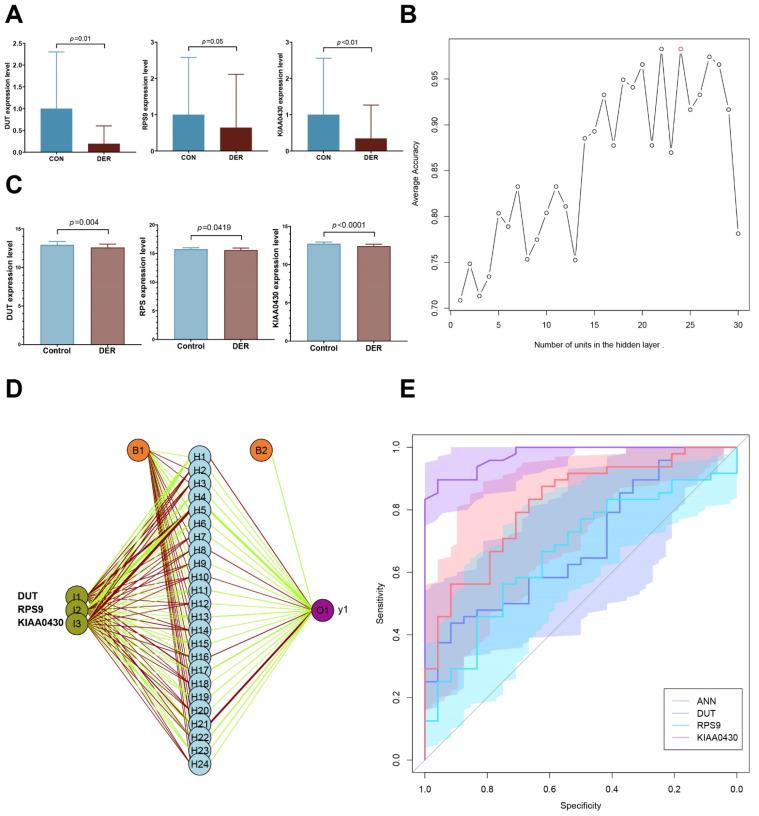
Artificial neural network prognostic model. (**A**) Validation of mRNA expressions of hub genes in DER (*n* = 10) and control (*n* = 9). (**B**) Average accuracy of each number of units. Twenty-four units exhibited the greatest accuracy (98.3%). (**C**) Expression levels of hub genes. (**D**) Schematic presentation of the ANN model for predicting DER. Dark green represents transmission of information from hub genes. Green and red colours represent positive and negative weights, respectively. Light blue represents the bias applied to hidden neurons. (**E**) ROC of ANN and single genes. AUCs of ANN, DUT, RPS9, and KIAA0430 were 0.975 (0.945–1), 0.688 (0.563–0.814), 0.665 (0.535–0.795), and 0.818 (0.716–0.919), respectively.

Before establishing the ANN model, first, we validated the altered mRNA expression levels of DUT (0.282 (0.104–3.547) vs. 0.039 (0.008–1.348)), RPS9 (0.452 (0.162–5.084) vs. 0.176 (0.044–4.81)), and KIAA0430 [0.5 (0.0417–4.953) vs. 0.0362 (0.009–2.955)] in endometrial tissues. Compared to the control group, the expressions of all three genes (*p* = 0.01, 0.05, and 0.008 for DUT, RPS9, and KIAA0430, respectively) in patients with defective endometrial receptivity were significantly downregulated (Figure 5A).

The deep machine learning algorithm (ANN) was used to evaluate the predictive power of selected hub genes for defective endometrial receptivity. First, the number of units in the hidden layer of the GSE58144 dataset was set. When the hidden layer was set to 24, the model achieved the highest prediction accuracy of 98.3% (Figure 5B).

The prognostic model was validated using the GSE165004 dataset. Expression levels of the three hub genes between the two groups were significantly different, consistent with results from the GSE58144 dataset (*p* = 0.004, 0.0419, and <0.001; Figure 5C). With regards to effect sizes of the three genes, DUT and RPS9 were moderate while KIAA0430 was large (Cohen’s d= −0.75, −0.52, and −1.25). Then, these hub genes were incorporated into the ANN prognostic model. Based on earlier findings, we set the number of units in the hidden layer to 24 and performed 100 cycles of simulation to improve the prognostic accuracy (Figure 5D). Prognostic results were averaged and plotted as ROC, and were found to compare to those from hub genes alone. As result, the AUC of the ANN module was 0.975 (95% CI 0.945–1), significantly better than that of DUT, RPS9, and KIAA0430 (*p* = 9.27 × 10^−6^, 1.15 × 10^−6^, and 5.33 × 10^−4^; Figure 5E). Sensitivity, specificity, and YI of the ANN model were 89.58 (95% CI 77.3–96.5), 95.83 (95% CI 78.9–99.9), and 0.854, respectively. For patients with unexplained infertility, the pregnancy success rate with assisted reproductive techniques is only 23–45% [25]. Based on prevalence, PPV and NPV were 97.7 (95% CI 86.3–99.7) and 82.1 (95% CI 66.6–91.4), respectively. All these results were superior to those of the other hub genes (Table 1).

## 4. Discussion

An altered immune microenvironment in the uterine cavity is an important factor in placental implantation, unexplained infertility, and first-trimester miscarriage. Abnormal endometrial immune cell infiltration has been reported in infertility. However, the significance of immune-related factors in assessment of endometrial receptivity has not been fully established. We found that the balance of the ratio between M1 and M2 macrophages is an important factor that affects endometrial receptivity for patients. Then, we screened different biological functional gene modules, obtained hub genes associated with M1/M2 macrophages and developed a machine deep learning prognostic model with an excellent predictive performance.

The balance of macrophage ratios is important for WOI and embryonic implantation in the endometrium. Factors that induce macrophage infiltration into the endometrium in mid-secretory phase, including chemokines, colony-stimulating factor (CSF)-1, and granulocyte macrophage-colony-stimulating factor (GM-CSF) are abundantly secreted by endometrial stromal cells in response to hormonal stimulation. Vascular endothelial growth factor receptor-1 (VEGFR-1) is involved in macrophage recruitment and angiogenesis at the implantation site. Macrophages have a high potential for plasticity and can modify their functions depending on changing microenvironments in tissues. Therefore, they are involved in different physiological functions and in disease development. Based on phenotypes and functions, macrophages can be classified into the activated M1 type and the alternatively activated M2 type. Interferon (IFN)-γ, lipopolysaccharide (LPS), or granulocyte macrophage-colony-stimulating factor induce macrophage maturation towards the M1 type, which activate Toll-like receptor signalling pathways, thereby playing a crucial role in clearance of residual fibres and tissue debris, as well as in the synthesis of pro-inflammatory cytokines and growth factors. In addition, M1 macrophages secrete various cytokines, including tumour necrosis factor alpha (TNF-α), interleukin (IL)-1α, and IL-6, which may be closely associated with the endometrial decidualization and embryonic implantation processes. Maturation of M2 macrophages can be induced by various cytokines, including IL-4, IL-13, glucocorticoids, as well as M-CSF/CSF1, and together with Tregs, they promote tissue remodelling and regeneration, wound healing, and anti-inflammation in the endometrial tissue. Altered expression levels of some cytokines, such as TNF-α and IL-1β, can affect the polarization shift between M1 and M2 macrophages, impacting reproductive outcomes. Therefore, the balance between M1 and M2 macrophages in the mid-secretory phase is crucial for the embryonic implantation process.

In this study, we found that the M1/M2 ratio was reduced in patients in the defective endometrial receptivity group, compared to the control group. The type of macrophage dominating the mid-secretory phase has not yet been established. Russell et al. [8] reported a significant increase in M2 macrophages during the luteal phase, which are thought to contribute to establishment of maternal immune tolerance to foetal antigens at the onset of implantation. However, Diao et al. [26] reported that the abundance of M2 macrophages was significantly low in the control group than in patients with failed embryonic implantation. Some studies have also suggested that, during implantation, activated M1 macrophages produce inflammatory cytokines and mediators, such as IL-6, IL-1β, TNF-α, and nitric oxide, inducing pro-inflammatory responses and promoting embryonic attachment to the decidua. These findings confirm the importance of the balance of macrophage polarization for embryonic implantation.

Although macrophage polarization is of great importance in pregnancy, their significance in reproductive prognosis has not been conclusively determined, mainly because of the subjectivity of cell counts by immunohistochemistry and difficulty with standardization. Moreover, immune cell infiltration dynamics are associated with the possibility of some systematic errors. Therefore, we used macrophage polarization-related factors as predictors for reproductive prognostic outcomes. To ensure that genes included in the prognostic model are representative of certain molecular functions or structures, gene selection was performed using the WGCNA approach. Analysis of gene modules associated with M1/M2 revealed that their functions are clustered in three main aspects of macrophage polarization in biological processes (antigen processing and presentation of exogenous peptide antigens via MHC class I, TAP−dependent, phagosome acidification, and interleukin-1-mediated signalling pathway). The main pathways of the modules were ribosomal, lysosomal and proteasomal pathways. Then, we selected representative genes (DUT, RPS9 and KIAA0430) in each gene module. In DER patients, these genes were suppressed, which were cross-corroborated using the GSE58144 as well as GSE165004 datasets and clinical samples. Their correlations with macrophages were further validated in the GSE5099 dataset, proving that their close associations are essential in homeostasis of macrophage polarization and maintenance of favourable endometrial receptivity (Graphical abstract). Specific mechanisms underlying the actions of the module and its hub genes on embryonic implantation in concert with endometrial macrophage polarization, in accordance with the relevant studies and enrichment analysis, are as follows.

In the blue module, the principal biological functions performed by the gene cluster are antigen processing and presentation of exogenous peptide antigens via MHC class I, TAP−dependent. MHC class I plays an intermediate role in regulation of macrophage phagocytosis. As part of the local immunity, macrophages recognize antigens on MHC class I and present them to T cells, which recognize the MHC-antigen complex through their T cell receptors that require additional costimulatory and cytokine signals. Therefore, macrophages can mediate and provide costimulatory signals and secrete cytokines required for effective T-cell activations [27]. M1 macrophages secrete various cytokines, including tumour necrosis factor alpha (TNF-α), interleukin (IL)-1α, and IL-6, which may be closely associated with endometrial decidualization and the embryonic implantation processes. Maturation of M2 macrophages can be induced by various cytokines, including IL-4, IL-13, glucocorticoids, as well as M-CSF/CSF1, and together with Tregs, they promote tissue remodelling and regeneration, wound healing, and exert anti-inflammatory effects in the endometrial tissue. Altered expression levels of some cytokines, such as TNF-α and IL-1β, can affect the polarization shift between M1 and M2 macrophage phenotypes, impacting reproductive outcomes. Meanwhile, class I expressions may be critical for avoidance of immunological rejection [28]. Its hub gene, RPS9, a component of the ribosomal 40s subunit, is an important factor in macrophage activation and polarization through its protein translation function. Several cytokines, such as IFN-γ, IL-10a, and IL-6 regulate ribosomes through receptors, thereby affecting macrophage metabolism as well as mRNA translation [29]. Pérez-Debén et al. [30] evaluated endometrial receptivity-related pathways and found that the ribosomal pathway is the most relevant for endometrial fertility. Furthermore, GSEA revealed a negative correlation between RPS9 and the proteasome. According to Fan et al. [31], molecules such as ribosomes in macrophages can be hydrolysed by the proteasome through ubiquitination. Inhibition of this pathway has been shown to have positive implications in protection of macrophages as well as the periphery from hypoxia-reoxygenation injury. In this study, RPS9 was significantly differentially expressed in IF and control groups for both GSE58144 and GSE165004 datasets. These findings imply that the module of antigen processing and presentation of exogenous peptide antigens via MHC class I in macrophage polarization play important roles in endometrial receptivity.

As for the turquoise module, the genes were enriched in the phagosome acidification biological process, which is directly associated with the phagocytic function of macrophages. DUT, as the hub gene, was enriched in lysosomal and ribosomal pathways by its related genes in the GSE5099 dataset, consistent with its module. Phagocytosis and antigen presentation by macrophages is dependent on phagosomal and lysosomal activities. Phagosome-like compartments containing antigens at some stages fuse with lysosomes to form phagolysosomes. Ariza et al. [32] reported that regulation of DUT in macrophages depends on their phagocytosis. DUT (dUTPase), an essential enzyme during nucleotide metabolism, hydrolyses dUTP to dUMP and pyrophosphate. Alternative splicing of this gene leads to different isoforms that are localized in the mitochondria or nucleus. dUTPase modulates innate immunity in human primary monocyte-derived macrophages through toll-like receptor (TLR) 2, leading to NF-kB activation and the production of pro-inflammatory cytokines. This process is achieved via macrophage phagocytosis of exosomes containing dUTPase. Then, interferon (IFN)-γ, lipopolysaccharide (LPS), or granulocyte macrophage-colony-stimulating factors induce macrophage maturation towards the M1 phenotype, which activates Toll-like receptor signalling pathways, thereby playing a crucial role in clearance of residual fibres and tissue debris, and in the synthesis of pro-inflammatory cytokines and growth factors. Wang et al. [32]. reported that DUT activation led to the production of large amounts of mtDNA, which bound the mitochondrial ribosomal proteins to co-synthesize mitochondria-associated proteins, further demonstrating the association between DUT and the ribosomal pathway. Therefore, enrichment analysis of the turquoise gene module in endometrial tissues and DUT in macrophage GSE5099 dataset revealed close associations among DUT, phagosome acidification, and the aforementioned two pathways, which affected the M1/M2 ratio in patients in the mid-secretory phase.

The green module’s biological function was enriched in interleukin-1-mediated signalling pathway, which is vital for macrophage functions and polarization. Elevated expression levels of cytokines, such as TNF-α in the early stages promote macrophage polarization, as well as the synthesis and secretion of IL-1 by macrophages. In addition, IL-1 in the early stages can act on interleukin-1-mediated signalling pathway to promote the synthesis and secretion of other cytokines that are intimately involved in regulation of the ribosomal pathway [33]. Its hub gene, KIAA0430, also known as meiosis regulator and mRNA stability factor 1 (MARF1), encodes a putative peroxisomal protein, which can silence targeted mRNA and inhibit gene expressions [34]. It can regulate the translational function of ribosomes as described above, consistent with the finding that it was enriched in the ribosomal pathway in the GSE5099 dataset. Meanwhile, we found that gene enrichment in the green module, where KIAA0430 is located, was closely associated with negative regulation of the proteasomal pathway. The negative correlation between KIAA0430 and the proteasomal pathway was verified in the macrophage GSE5099 dataset. Proteasomes are protein disruption devices that are involved in many essential cellular functions, such as cell cycle regulation, cell differentiation, signal transduction pathways, antigen processing for proper immune responses, stress signalling, inflammatory responses, and apoptosis. Moreover, they are involved in macrophage polarization. Cytokines such as IFN-γ and TNF-α regulate macrophage functions by moderating the proteasomal pathway. Han et al. [35] documented that proteasomes in endometrial stromal cells can mediate diminished protein stabilities of HOXA10, a histone that is important for promoting endometrial decidualization, leading to defective endometrial receptivity. In contrast, negative regulation of the proteasomal pathway by KIAA0430 facilitates the maintenance of favourable endometrial receptivity.

In this study, we used the ANN approach to establish a prognostic model. Traditional models were often constructed through the linear approach. Model optimization requires the inclusion of factors that are both normally distributed and independent. The ANN model, however, can weigh each feature variable according to its importance and then perform the summation of activity function, thus, it requires fewer factors to provide a more accurate classification. Although we found an imbalance between M1 and M2 macrophages, we performed the diagnosis by expression of factors in different gene modules instead of direct microscopic counts to improve reproducibility of the results and reduce measurement errors associated with subjectivity. The predictive accuracy of the model in the GSE58144 dataset was up to 98.3%, while its AUC was 0.975 in the validation GSE165004 dataset, which were significantly better than the predictive method with a single factor. Its performance was also superior to those of conventional endometrial receptivity examinations, such as ultrasound for endometrial thickness (>7 mm) with a sensitivity of 99% and a specificity of 3% [2]. The significance of this predictive method is that it effectively determines the receptive status of the endometrium. Jena et al. [4] reported that there is a dynamic balance of macrophage polarization patterns in the endometrium in response to the menstrual cycle, suggesting that this model is appropriate for assessing endometrial receptivity in patients with RIF due to WOI. Importantly, uterine cavity procedures (induced abortion, adhesiolysis, or polypectomy), inflammatory responses and transient immune dysregulation (dysbacteriosis or endometritis) due to stress can lead to embryonic implantation failure [36,37]. However, this model is highly correlated with the immune environment and is appropriate for informing optimal timing of natural or IVF pregnancies in such patients. For patients with unexplained infertility, assessment of endometrial receptivity using this tool is beneficial in identifying the cause and predicting the immediate outcome of IVF. In addition, for patients subjected to hysteroscopic procedures (such as those with polyps, submucous myomas, uterine adhesions) but who desire fertility, this assessment is equally crucial. For patients with poor endometrial receptivity, especially the elderly, it may not be advisable to continue expectant management (spontaneous pregnancy) after the procedure. Simultaneously, for patients without strong fertility, poor endometrial receptivity tends to indicate the potential need for several IVF treatments and a higher risk of failure [25], which may also provide them with some reference values for benefit-risk assessment in view of the high cost of treatment.

This study preliminarily validated the changes in the ratio between M1 and M2 macrophages and established a prognostic model, however, there are some limitations. First, other assays, such as qRT-PCR or ELISA, were not performed to assess the model, which should be validated through further large sample experiments. Second, for the mechanistic study, only enrichment analysis was performed, which are all based on mRNA expression levels. Therefore, it was not possible to observe the effects of changes in translational or post-translational protein levels for mechanistic prediction, which should be validated further. Importantly, immunofluorescence results and cross-validation between multiple data sets guarantees reliability of the mechanism as well as the ANN model. In short, based on our results, the consistency of validation, as analysed by different methods and datasets, was mutually confirmed.

## 5. Conclusions

The balance between M1 and M2 macrophages is essential for the pregnancy process. Gene modules associated with biological processes of antigen processing and presentation of exogenous peptide antigens via MHC class I, TAP−dependent, phagosome acidification, and the interleukin-1-mediated signalling pathway can impact macrophage polarization, thereby ameliorating endometrial receptivity. Furthermore, the established ANN model based on hub genes can effectively assess endometrial receptivity to predict reproductive outcomes for patients and inform individualized clinical management strategies.

## Figures and Tables

**Table 1:** Diagnostic efficacies of the prognostic model and single genes.

Methods	Sensitivity	Specificity	YI	PPV	NPV
ANN	89.58 (77.3–96.5)	95.83 (78.9–99.9)	0.8542	97.7 (86.3–99.7)	82.1 (66.6–91.4)
DUT	43.75 (29.5–58.8)	91.67 (73.0–99.0)	0.3542	91.3 (72.8–97.6)	44.9 (38.2–51.8)
RPS9	56.25 (41.2–70.5)	75 (53.3–90.2)	0.3125	81.8 (68.3–90.4)	46.2 (36.6–56.0)
KIAA0430	79.17 (65.0–89.5)	70.83 (48.9–87.4)	0.5	84.4 (74.1–91.1)	63 (48.1–75.7)

Abbreviations: YI Youden index, PPV positive predictive value, NPV negative predictive value.

## Data Availability

The raw data supporting the conclusions of this article will be made available by the authors, without undue reservation.

## Supplementary Materials

The following are available online at https://www.mdpi.com/article/10.3390/vaccines10020139/s1, Table S1: Overview of details for original studies, Table S2: Overview of the demographics and other characteristics of the recruited patients, Table S3: Specific primers used in qRT-PCR analysis.

## Abbreviations

WOI: window of implantation; RIF: repeated implantation failure; NK: Natural killer; WGCNA: weighted gene co-expression network analysis; ANN: artificial neural network; DER: decreased endometrial receptivity; RMA: Robust multi-array average; BSA: bovine serum albumin; PBS: phosphate-buffered saline; GO: Gene Ontology; BP: Biological Process; CC: Cellular Component; MF: Molecular Function; KEGG: Kyoto Encyclopedia of Genes and Genome; GSEA: Gene-set enrichment analysis; qRT-PCR: Quantitative Real-Time PCR Analysis; ROC: receiver operating characteristic; PPV: positive predictive value; NPV: negative predictive value; MS: module significance; CSF-1: colony-stimulating factor; GM-CSF: granulocyte macrophage-colony-stimulating factor; VEGFR-1: Vascular endothelial growth factor receptor-1; IFN-γ: Interferon; LPS: lipopolysaccharide; TNF-α: tumor necrosis factor alpha; IL-1α: interleukin.
