# Supplementary material for: Establishment of an Artificial Neural Network Model Using Immune-Infiltration Related Factors for Endometrial Receptivity Assessment
Source: Vaccines (Basel). 2022 Jan 18;10(2):139. doi: 10.3390/vaccines10020139 (PMC8875905; doi:10.3390/vaccines10020139)
Supplement: Supplementary file 1 [file vaccines-10-00139-s001.zip › vaccines-1502093-supplementary.pdf]

Supplementary Table S1. Overview of details for original studies.

GEO database	Region	Platform	Description	Sample size
GSE58144	Endometrium	GPL15789	Between 2006 and 2013, mid-luteal phase endometrial biopsies were taken in the natural cycle from two serial cohorts, each consisting of women with RIF, defined as ≥3 failed IVF/ICSI treatments or replacement ≥10 embryos without the occurrence of pregnancy and controls. All subjects had undergone IVF/ICSI treatment in two tertiary hospitals. The control group had all conceived within the first two cycles of ICSI or first three cycles of IVF treatment, and were therefore considered unlikely to have an endometrial factor. Microarray profiling and subsequent analysis sought to identify a gene expression signature predictive of RIF.	43(RIF) vs. 72(CON)
GSE5099	Macrophage	GPL96	Monocytes were induced to mature to macrophages with M-CSF. Cells were then activated with Interferon gamma and LPS or IL-4.	3(M1) vs. 3(M2)
GSE165004	Endometrium	GPL16699	All patients involved in this prospective cohort study were recruited from Istanbul University School of Medicine between August 2014 and August 2015. Three cohorts (fertile controls, patients with RPL and UI) were studied. None of the patients has received a prior infertility treatment and were not under a current treatment. The first cohort comprised fertile control patients who presented to our gynecology department for well woman examinations. The inclusion criteria were regularly cycling women aged under 35 years with at least one live birth, no history of infertility/treatment, no previous miscarriages and no associated gynecologic (endometriosis, fibroids, active or history of pelvic inflammatory disease) or other medical comorbidities (hyperprolactinemia, thyroid disease etc). The remaining cohorts constituted patients who presented to our in vitro fertilization (IVF) unit. The second cohort included patients with RPL with no history of successful	48(RPL and UI) vs. 24(CON)

pregnancies. The inclusion criteria for this group were regularly cycling women aged under 35 years with at least two consecutive pregnancy losses of 20 weeks or less, normal follicle-stimulating hormone (FSH), luteinizing hormone (LH), estradiol (E2), prolactin (PRL), and thyroid-stimulating hormone (TSH) levels at day 2-3, normal uterine cavity shape and size, and bilateral tubal patency observed on hysterosalpingogram, no mutations detected in Factor V (Leiden) and prothrombin gene analysis, normal antithrombin III, protein C and S activity, negative results for lupus anticoagulant evaluation, cardiolipin antibody (IgM and IgG), and beta2-glycoprotein antibody (IgM and IgG) and normal karyotype. Their partners have normal spermiogram results and normal karyotype. The third cohort was formed by women with UI at least of 18 months of duration. The inclusion criteria for this group were regularly cycling women aged under 35 years with normal FSH, LH, E2, PRL, and TSH levels at day 2-3, normal uterine cavity shape and size, and bilateral tubal patency observed on a hysterosalpingogram. Their partners have normal spermiogram results.

Supplementary Table S2. Overview of the demographics and other characteristics of the recruited patients.

Parameter	DER<br>N=13	Control<br>N=10	*P*
**Age, mean (SD)**	34.75 (3.92)	34.1 (3.39)	*P* = 0.71
**BMI, mean (SD)**	20.88 (6.75)	23.1 (3.62)	*P* =0.25
**Length of menstrual cycle, median (range)**	28 (25, 42)	27 (23, 38)	*P* = 0.68
**Gravidity, median (range)**	1 (0, 3)	1 (0, 3)	*P*=0.95
**Parity, median (range)**	1 (0, 1)	0 (0, 2)	*P*=0.51
**PBAS, median (range)**	43 (15, 182)	51.5 (33, 112)	*P*= 0.90
**Dysmenorrhoea, median (range)**	0 (0, 6)	0 (0, 4)	*P* = 0.86
**Previous uterine procedure, median (range)**	2 (1, 4)	1 (1, 3)	*P*= 0.11

Abbreviations: PBAC, pictorial blood loss assessment chart.

Supplementary Table S3. Specific primers used in qRT-PCR analysis.

Gene		Sequence (5′-3′)
**DUT**	Forward	5′-GGTGATCGAATTGCACAGCTC-3′
	Reverse	5′-TGAACCCCTTTCGGTGTCATC-3′
**RPS9**	Forward	5′-GAAATCTCGTCTCGACCAAGAG-3′
	Reverse	5′-GGTCCTTCTCATCAAGCGTCA-3′
**MARF1 (KIAA0430)**	Forward	5′-ACCCTCCACTTCGCCAATG-3′
	Reverse	5'-CTTTGCGAGTCTAACAGTGCG-3′
**ACTB**	Forward	5′-CATGTACGTTGCTATCCAGGC-3′
	Reverse	5′-CTCCTTAATGTCACGCACGAT-3′
